# Identifying SARS-COV-2 infected patients through canine olfactive detection on axillary sweat samples; study of observed sensitivities and specificities within a group of trained dogs

**DOI:** 10.1371/journal.pone.0262631

**Published:** 2022-02-14

**Authors:** Dominique Grandjean, Capucine Gallet, Clothilde Julien, Riad Sarkis, Quentin Muzzin, Vinciane Roger, Didier Roisse, Nicolas Dirn, Clement Levert, Erwan Breton, Arnaud Galtat, Alexandre Forget, Sebastien Charreaudeau, Fabien Gasmi, Caroline Jean-Baptiste, Sebastien Petitjean, Katia Hamon, Jean-Michel Duquesne, Chantal Coudert, Jean-Pierre Tourtier, Christophe Billy, Jean-Marc Wurtz, Anthony Chauvin, Xavier Eyer, Sabrina Ziani, Laura Prevel, Ilaria Cherubini, Enfel Khelili-Houas, Pierre Hausfater, Philippe Devillier, Loic Desquilbet

**Affiliations:** 1 Ecole Nationale Vétérinaire d’Alfort (Alfort School of Veterinary Medicine), University Paris-Est, Maisons-Alfort, France; 2 Université Franco-Libanaise St Joseph (Saint Joseph University of Beirut), Beirut, Lebanon; 3 Service Départemental d’Incendie et de Secours de l’Oise (Fire and Rescue Service), Tillé, France; 4 Service Départemental d’Incendie et de Secours des Yvelines (Fire and Rescue Service), Versailles, France; 5 Hôpital d’Instruction des Armées Begin (Begin Military Hospital), Saint-Mandé, France; 6 Centre Hospitalier François Quesnay (François Quesnay Hospital Centre), GHT Yvelines, Mantes-la-Jolie, France; 7 Site d’Altkirch GHRMSA (Groupement Hospitalier Mulhouse Sud Alsace), Altkirch, France; 8 Hôpital Lariboisière APHP (Lariboisière Hospital, APHP Great Paris Hospitals), Paris, France; 9 Hôpitaux de Saint-Maurice (Saint-Maurice Hospital), Saint-Maurice, France; 10 Hôpital Foch (Foch Hospital), Suresnes, France; 11 Hôpital Pitié-Salpêtrière APHP (Pitié-Salpêtrière Hospital, APHP Great Paris Hospitals), Paris, France; 12 Ecole nationale vétérinaire d’Alfort, Univ Paris Est Créteil, INSERM, IMRB, Maisons-Alfort, France; Stanford University School of Medicine, UNITED STATES

## Abstract

There is an increasing need for rapid, reliable, non-invasive, and inexpensive mass testing methods as the global COVID-19 pandemic continues. Detection dogs could be a possible solution to identify individuals infected with SARS-CoV-2. Previous studies have shown that dogs can detect SARS-CoV-2 on sweat samples. This study aims to establish the dogs’ sensitivity (true positive rate) which measures the proportion of people with COVID-19 that are correctly identified, and specificity (true negative rate) which measures the proportion of people without COVID-19 that are correctly identified. Seven search and rescue dogs were tested using a total of 218 axillary sweat samples (62 positive and 156 negative) in olfaction cones following a randomised and double-blind protocol. Sensitivity ranged from 87% to 94%, and specificity ranged from 78% to 92%, with four dogs over 90%. These results were used to calculate the positive predictive value and negative predictive value for each dog for different infection probabilities (how likely it is for an individual to be SARS-CoV-2 positive), ranging from 10–50%. These results were compared with a reference diagnostic tool which has 95% specificity and sensitivity. Negative predictive values for six dogs ranged from ≥98% at 10% infection probability to ≥88% at 50% infection probability compared with the reference tool which ranged from 99% to 95%. Positive predictive values ranged from ≥40% at 10% infection probability to ≥80% at 50% infection probability compared with the reference tool which ranged from 68% to 95%. This study confirms previous results, suggesting that dogs could play an important role in mass-testing situations. Future challenges include optimal training methods and standardisation for large numbers of detection dogs and infrastructure supporting their deployment.

## Introduction

As the global COVID-19 pandemic continues, there is an ever-present need for fast, reliable, and inexpensive testing methods.

One possible solution involves using detection dogs to identify individuals infected with SARS-CoV-2. SARS-CoV-2 generates signature volatile organic compounds (VOC) which are volatile at ambient temperatures, and may be detectable by dogs in breath, urine, tears, saliva, faeces and sweat. VOCs emanating from the skin contribute to an individual’s body odour and may convey information about metabolic processes [1,2]. For many years, it has been suggested that VOCs could revolutionise non-invasive medical diagnostics in humans. Several studies have demonstrated positive results using dogs for early detection of colorectal cancer [3,4], and encouraging results for lung cancer [5–8], melanoma [9,10], prostate cancer [11–13] and liver cancer [14]. Detection dogs are commonly used to identify hypoglycaemic episodes in diabetic patients [15–18] and epileptic fits [19]. Canine olfactory detection is also expanding for infectious diseases. In 2014, Aksenov et al. [20,21] found the VOCs produced by B-lymphocyte cultures infected by avian H9N2, H6N2 and human H1N1 influenza viruses were unique and specific to each viral subtype. Recently, Abd El Qader et al. [22] showed cell cultures infected by bacterial and viral species produced specific VOCs.

It was hypothesised that COVID-19 would produce a specific VOC resulting in the Nosaïs-COVID-19 program being launched by the Alfort School of Veterinary Medicine (France) and the Saint-Joseph University of Beirut (Lebanon) in March 2020. It aims to train dogs to detect SARS-CoV-2 carriage in asymptomatic or subclinical individuals using axillary sweat samples, under controlled conditions. An initial proof-of-concept study led by our team [23] provided evidence that detection dogs could identify a specific scent in COVID-19 patient sweat samples tested positive using the Pasteur Institute Reverse Transcription Polymerase Chain Reaction (RT-PCR) SARS-CoV-2-IP24 technique, recommended by the World Health Organisation [24,25]. The success rate ranged from 84% to 100%. Articles from other teams following this proof-of-concept study, showed similar results with saliva [26], respiratory secretions [27,28] and urine samples [29].

This study is the project validation stage and aims to establish sensitivity and specificity values for canine detection of COVID-19.

## Materials and methods

This research is the COVIDOG ancillary study to the COVIDEF study (Cohort of patients infected by the virus SARS-CoV-2 or suspected to be infected), promoted by Assistance Publique Hôpitaux de Paris (Parisian hospitals) and led by Professor Hausfater.

### Patient recruitment

COVID-19 positive and negative patients were recruited from 13 centres including hospitals, COVID-19 screening centres, and fire departments around Paris, France. Patients presenting to one of these participating centres with COVID-19 clinical symptoms (fever, cough, sore throat, fatigue, or body aches), and having tested positive for SARS-CoV-2 using a RT-PCR or PCR test were included as positive individuals. COVID-19 positive individuals were excluded if they had received a medical treatment for more than 36 hours prior to sampling to prevent interference from long-term medical treatments in the sample.

Matched COVID-19 negative controls (medical staff or inpatients) were recruited from the same centre as each COVID-19 positive participant to avoid confounding with background hospital odour [30,31].

All individuals meeting these inclusion criteria were invited to participate in the study and signed an individual informed consent form approved by the national ethics committee.

### Samples

Trained health care professionals collected axillary sweat samples from all participants without contaminating them with their own scent. Each hospital used their own gloves which were identical for sampling COVID-19 positive and negative patients. Sufficient samples were obtained for training and the validation steps. Sweat sample choice, sampling site, sampling method, and biological safety measures have been previously published [23].

### Patient data collection

Demographic and medical data were collected for each recruited patient, included age, gender, clinical signs (dyspnoea, fatigue, fever, dry cough, muscular pain, headache, loss of smell, loss of taste, diarrhoea, nasal discharge, colic and migraine), medical history (including hypertension, diabetes, obesity, and arthrosis), and concomitant medication at the time of sampling (analgesics, anti-coagulants, anti-hypertensive drugs, anti-inflammatory drugs, antibiotics, antacids, anti-diabetic drugs, statins, bronchodilators, antidepressants, thyroid hormones, anxiolytics, and spasmolytics).

### Canine resources and initial training

**Fig 1:**
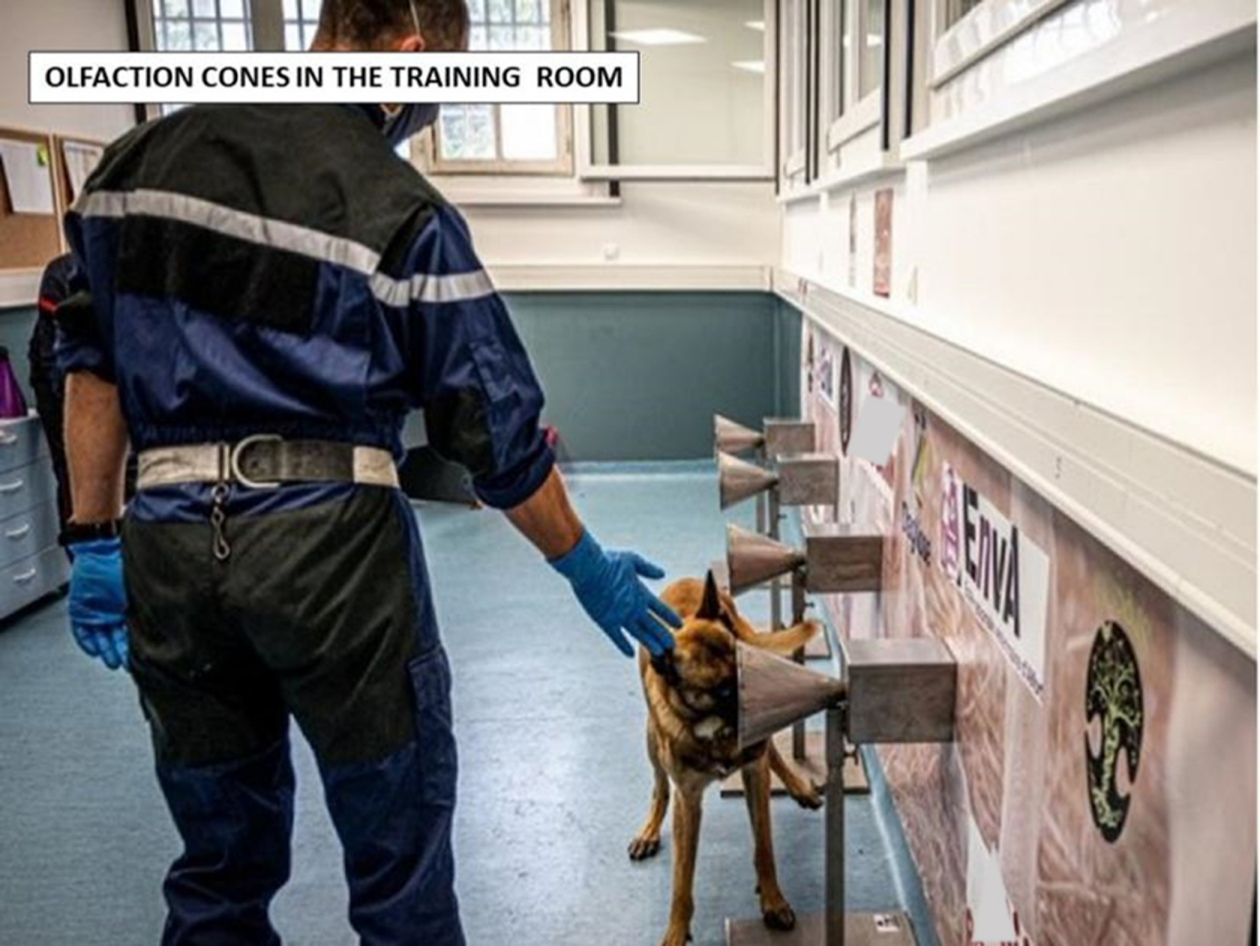
Olfaction cone line in the training room.

**Fig 2:**
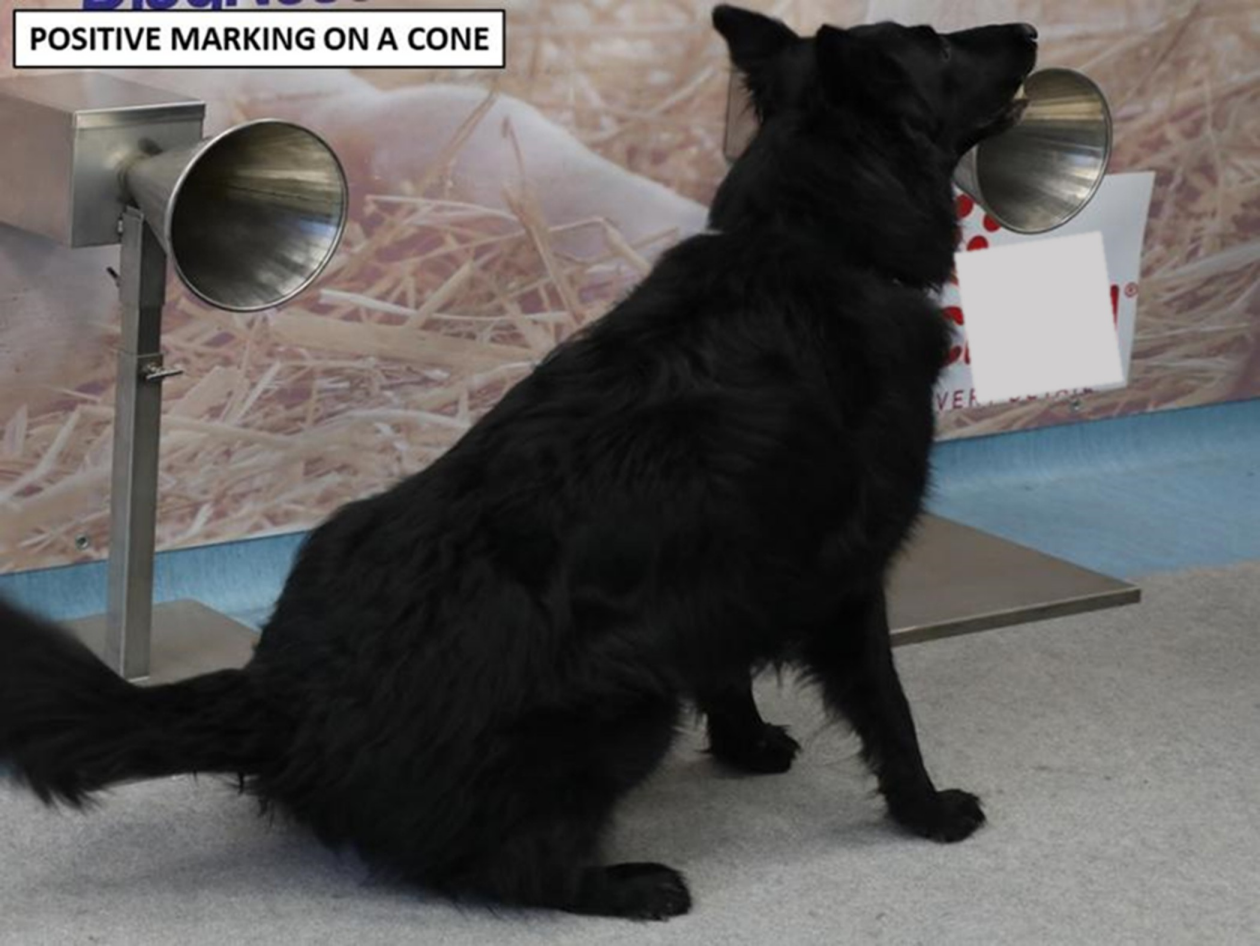
Dog showing a positive alert response in front of a cone containing a positive sample.

Seven search and rescue dogs trained to seek human scent (Table 1) from two French fire departments were trained for this study. The 8-week training course followed the protocol developed in our previous publication [23], with lines of olfaction cones (Fig 1), positive alert response by sitting in front of the cone (Fig 2) and positive reinforcement.

**Table 1:** Characteristics of the seven COVID-19 detection dogs.

Name	Gender	Breed	Age (years)	Organisation	Speciality
Leyko	M	Malinois	5	SDIS78*	SAR****
Jinko	M	Groenendael	6	SDIS78	SAR
Ortie	F	Malinois	2.5	SDIS78	SAR
Oska	F	Malinois	2.5	SDIS78	SAR
Oxmo	M	Malinois	2.5	SDIS78	SAR
Ouija	M	Dutch Shepherd	2	ENVA**	None
Joye	F	Malinois	6	SDIS60***	SAR

* Service Départemental d’Incendie et de Secours 78 (Fire and Rescue Service 78).

** Ecole Nationale Vétérinaire d’Alfort (Alfort School of Veterinary Medicine).

*** Service Départemental d’Incendie et de Secours 60 (Fire and Rescue Service 60).

**** Search and Rescue Dog.

Training started on September 14, 2020 for the first dog and ended on November 6, 2020 for the last dog. The training process followed a five-step procedure:

1. Learning line work with olfaction cones
2. Imprinting (memorisation) COVID-19 sample odour (positive samples and empty cones in the line)
3. Introduction of mocks (positive sample and clean swabs in the line)
4. Introduction of negatives with no mocks in the line
5. Removing all positive samples (only negative samples in the line)

Handlers judged when each dog was ready for the validation testing session. All seven dogs were deemed trained and ready for the testing session after the 8 weeks.

Dog welfare was fully respected, with toy rewards and no induced physical or mental fatigue. A total of 106 positives and 242 negative samples were used during training.

### Biological safety of dogs and humans

**Fig 3:**
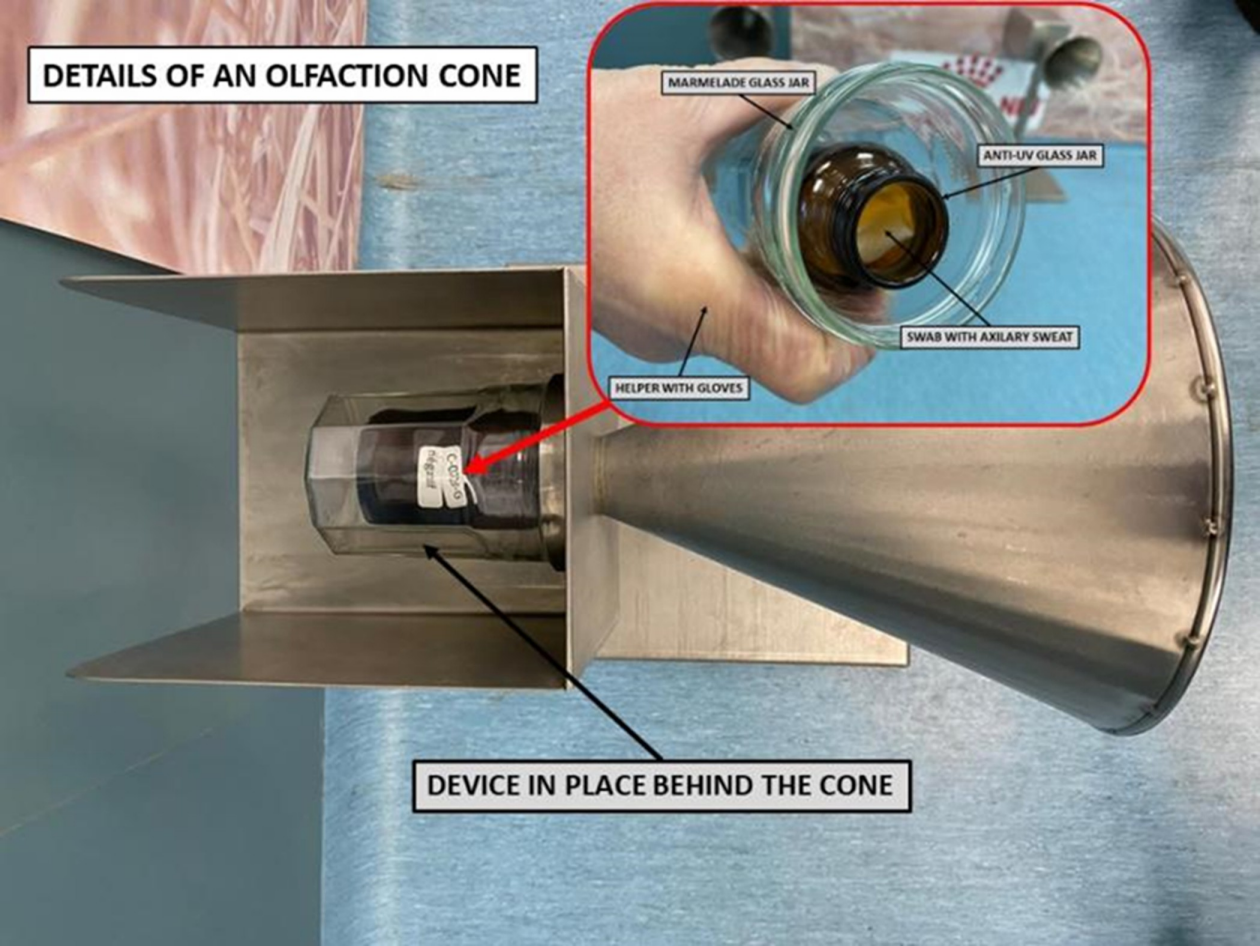
Olfaction cone with double sample protection and no possibility of direct contact with the dog.

We assumed that like SARS-CoV-1, SARS-CoV-2 would not survive longer than several hours on cotton [32]. For safety reasons, samples (stored at +4°C) were not used for training or testing sessions within 24 hours of collection. The olfaction cone prevented dogs from coming in direct contact with the samples (Fig 3).

### Testing protocol

**Fig 4:**
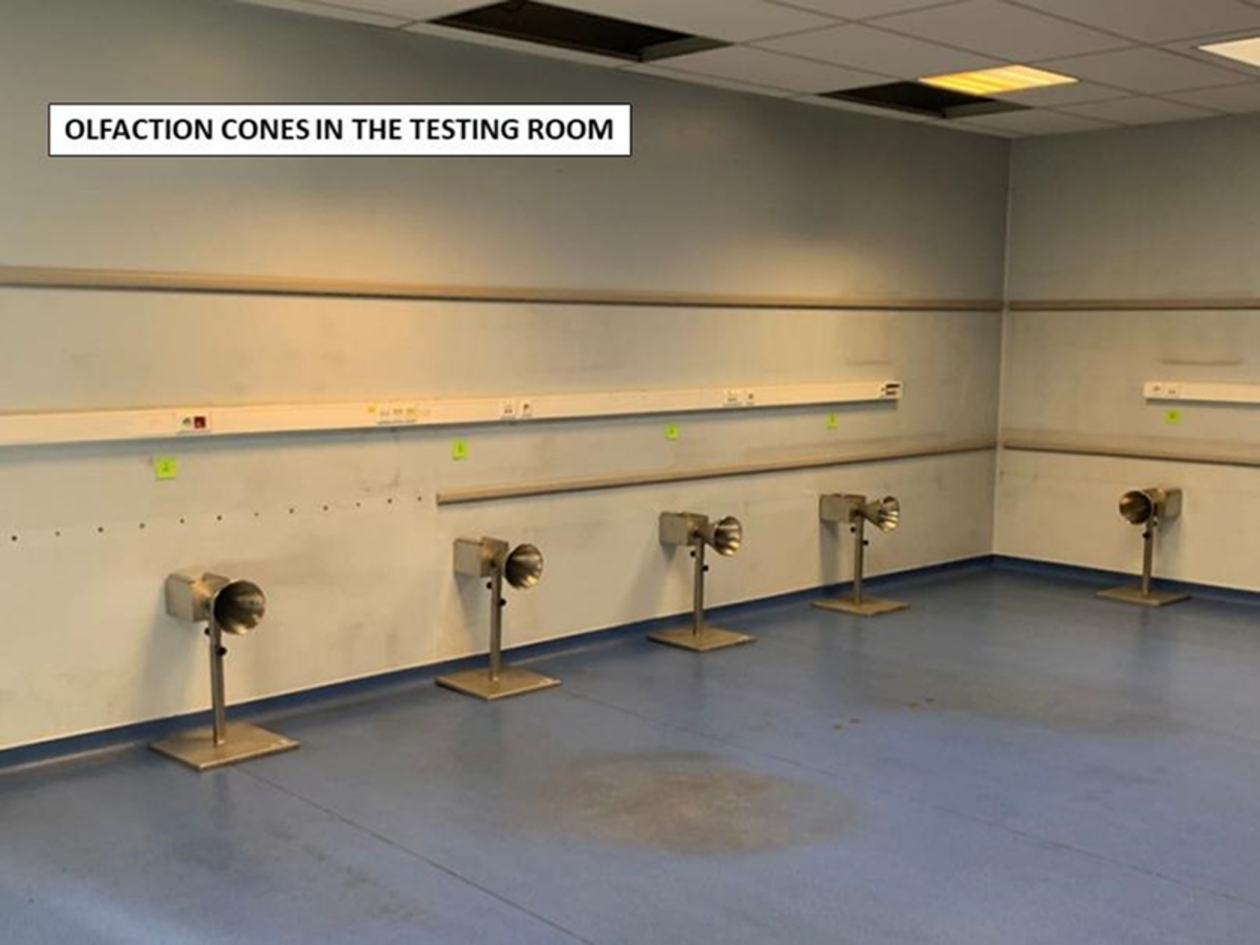
Testing room with its olfaction cones.

The testing sessions took place in the Poissy Fire Department where lines of five to eight cones were set up in a dedicated room (Fig 4).

Each cone contained either a COVID-19 negative or positive sample. The number of positive and negative samples and their position in the line were randomised using a dedicated website and double-blinded to the handler, dog and data-recorder, as recommended by Johnen [33]. This randomised sample distribution ensured that each line contained at least one positive and one negative COVID-19 sample. The number of COVID-19 positive and negative samples used for the 30 testing session lines are shown in Table 2.

**Table 2:** Characteristics of the 30 lines used for testing sessions.

Characteristics	Overall (n = 30)
Number of cones, n (%)	
5 cones	6 (20)
6 cones	2 (7)
8 cones	22 (73)
Number of COVID-19 negative samples in the line, n (%)	
3	3 (10)
4	6 (20)
5	6 (20)
6	12 (40)
7	3 (10)
Number of COVID-19 positive samples in the line, n (%)	
1	6 (20)
2	17 (57)
3	6 (20)
4	1 (3)

**Fig 5:**
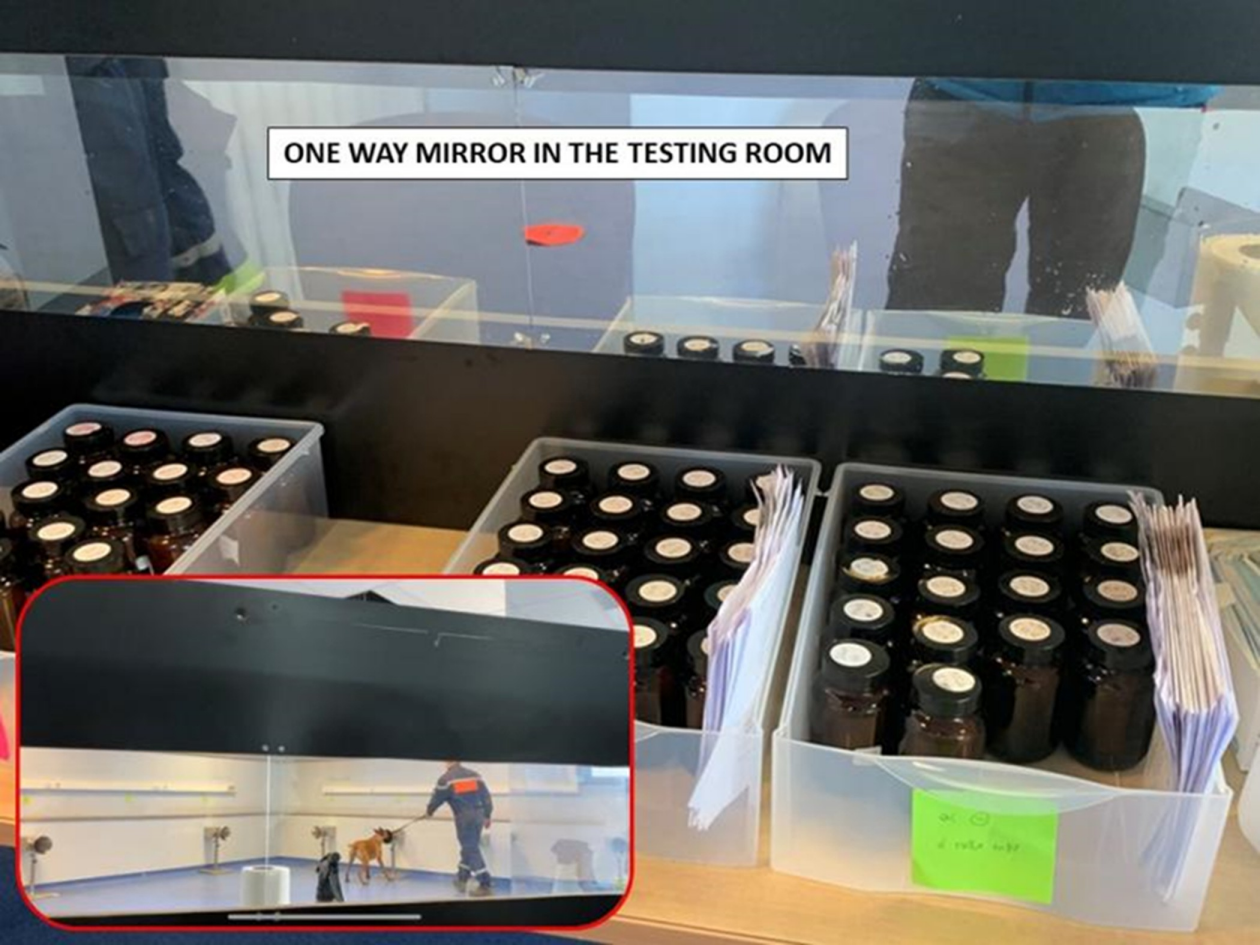
One way glass hiding the data recorder in the testing room.

A dedicated person placed the samples while the room was empty, and then immediately left the room without any contact with other investigators or handlers. For each new sample placement, this person wore new disposable gloves (same brand throughout the testing sessions) and a mask to prevent contaminating the olfactive environment. After sample placement, the data recorder sat behind one-way glass so they could not be seen by the dog or handler (Fig 5).

The dog entered the room with their handler and sniffed each cone, one by one. When the dog thought the cone contained a positive sample, a positive alert response was given such as sitting, barking, or scratching in front of the concerned cone. The handler then rewarded the dog and announced the positive alert response to the data recorder. The handler then asked the dog to resume the task for the remaining cones in the line (sequential line). Once all dogs had completed the line, the dog handlers were informed of the COVID-19 positive sample(s) location or absence.

Six dogs each completed 30 lines and one dog completed 28 as it was required for an emergency. Once a dog had sniffed all the cones in the line, the pair left the room, and the data recorder cleaned the cones with water to remove traces left by the previous dog. The next dog then entered the room and sniffed the cones in the same line.

Once the seven dogs had completed the line, the cones were cleaned with 3% concentrated acetone solution. New randomised samples were placed in the cones ten minutes after cleaning and a new trial cycle could start.

None of the samples used for the testing sessions had ever been used during the training sessions, and no dog ever sniffed a sample more than once.

### Statistical analysis

Statistical analyses were performed using SAS® University Edition (SAS Institute Inc., Cary, NC, USA). Sensitivity was calculated for each dog by dividing the number of COVID-19 positive samples the dog correctly identified by the total number of COVID-19 positive samples the dog sniffed during the testing sessions. Specificity was calculated for each dog by dividing the number of COVID-19 negative samples the dog correctly identified by the total number of COVID-19 negative samples the dog sniffed during the testing sessions.

To remove potential confounding bias, sensitivity and specificity were also calculated separately for samples from:

- Male participants
- Female participants
- Individuals under 50 years of age
- Individuals over 50 years of age
- Participants recruited from Foch and Rambouillet hospitals (hospitals with the highest positive participant recruitment)
- Individuals not presenting the most frequently reported clinical signs (dyspnoea, fatigue, fever, dry cough, and muscular pain)
- Individuals without the most frequently reported past or current medical conditions (hypertension and diabetes)
- Individuals not treated with the most frequently reported drugs (analgesics, anti-coagulants, anti-hypertensive drugs, anti-inflammatory drugs, and antibiotics).

The 95% confidence interval (CI) was calculated using Jeffreys’ method [34].

Positive and negative predictive values were calculated for each dog using their observed sensitivity and specificity and according to five infection probability scenarios reflecting the risk of an individual being SARS-CoV-2 positive [35,36]. These infection probabilities ranged from 10% to 50% and were based on clinical symptoms and SARS-Cov-2 prevalence in the area where the patient lives and works. This range covers the expected range for settings where COVID-19 detection dogs are likely to work (such as airports or mass events). Positive and negative predictive values are also presented for a reference diagnostic tool with 95% sensitivity and specificity as a comparison.

Binary and qualitative variables were presented as numbers and proportions, and the quantitative variable (age) was presented as medians and interquartile ranges.

### Ethics

This study was conducted in strict accordance with the Guide for the Care and Use of Animals recommendations (articles R214-87 to R214-137 of the rural code), updated by decree 2013–118 and five decrees edited on February 1, 2013. The Alfort School of Veterinary Medicine animal experiment ethics committee) and the Ile-de-France Protection of Persons Committee approved the protocol on March 30, 2020. It also follows the French Public Health Code (article L1121-1/2).

## Results

A total of 62 COVID-19 positive patients and 156 COVID-19 negative patients were recruited for this study, producing 218 sweat samples used in testing sessions. The proportion of females was slightly higher among COVID-19 negative patients (50%) compared with COVID-19 positive patients (44%; Table 3).

**Table 3:** Baseline characteristics of the 218 COVID-19 positive and negative patients.

Variables	Overall (n = 218)	COVID-19 negative (n = 156)	COVID-19 positive (n = 62)
Female, n (%)	100 (46)	69 (44)	31 (50)
Age (years)*	51 [37; 70]	50 [37; 70]	56 [40; 69]
Hospital, n (%)			
Foch	36 (17)	17 (11)	19 (31)
Rambouillet	34 (16)	24 (15)	10 (16)
Houilles	32 (15)	27 (17)	5 (8)
Other hospitals	82 (38)	60 (28)	22 (35)
Missing data	34 (16)	28 (18)	6 (10)
Most frequently reported clinical signs, n (%)			
Dyspnoea	41 (19)	8 (5)	33 (53)
Fatigue	35 (16)	6 (4)	29 (47)
Fever	31 (14)	4 (3)	27 (44)
Dry cough	30 (14)	4 (3)	26 (42)
Muscular pain	27 (12)	8 (5)	19 (31)
Most frequently reported past or current diseases, n (%)			
Hypertension	57 (26)	36 (23)	21 (34)
Diabetes	29 (13)	13 (8)	16 (26)
Current treatments, n (%)			
Analgesics	42 (19)	30 (19)	12 (19)
Anti-coagulant	32 (15)	12 (8)	20 (32)
Anti-hypertensive drugs	28 (13)	21 (13)	7 (11)
Anti-inflammatory drugs	23 (11)	11 (7)	12 (19)
Antibiotics	22 (10)	8 (5)	14 (23)

*Median [interquartile range].

Age distribution was similar between the two groups (medians of 50 years old for COVID-19 negative patients and 56 years old for COVID-19 positive patients). Patients were recruited mainly from Foch hospital (17%), Rambouillet hospital (16%), and Houilles Rescue Centre (15%; Table 3). Hospital data were missing for 16% of the recruited patients, mainly because recruitment was from many hospitals where samplers did not complete the requested documents correctly. The most frequently reported clinical signs were dyspnoea (19%), fatigue (16%), fever (14%), dry cough (14%) and muscular pain (12%). The most frequently reported past or current medical conditions were hypertension (26%) and diabetes (13%). Each of these clinical signs and medical conditions were more frequent among COVID-19 positive patients than negative patients. The most frequently reported drugs used were analgesics (19%), anti-coagulants (15%), anti-hypertensive drugs (13%), anti-inflammatory drugs (11%), and antibiotics (10%; Table 3).

The overall sensitivity was equal or higher than 87% for all but one dog (Ortie), where sensitivity was 60%. The overall specificity was equal or higher than 85% for all but one dog (Joye), where specificity was 78% (Table 4).

**Table 4:** Overall sensitivities and specificities of the seven dogs calculated from the 218 patients.

Dog	n+	N+	Overall Se (95% CI)	n-	N-	Overall Sp (95% CI)
Leyko	54	62	87 (77–94)	144	156	92 (87–96)
Jinko	55	62	89 (79–95)	135	156	87 (81–91)
Ortie	37	62	60 (47–71)	141	156	90 (85–94)
Oska	53	58	91 (82–97)	122	144	85 (78–90)
Oxmo	58	62	94 (85–98)	142	156	91 (86–95)
Ouija	56	62	90 (91–96)	142	156	91 (86–95)
Joye	55	62	89 (79–95)	122	156	78 (71–84)

n+: Number of COVID-19 positive samples the dog correctly identified; N+: Total number of COVID-19 positive samples sniffed by the dog; n-: Number of COVID-19 negative samples the dog correctly identified; N-: Total number of COVID-19 negative samples sniffed by the dog; Se: Sensitivity; Sp: Specificity; CI: Confidence interval.

Sensitivity remained virtually the same after stratification for patient gender, age (≤50 years old versus > 50 years old), recruitment from Foch or Rambouillet hospitals, absence of the most frequently reported health conditions, and no use of the most frequently reported drugs (Tables 5 and 6).

**Table 5:** Sensitivities and specificities for the seven dogs with the 218 patients stratified according to sex and age, and for patients recruited from Foch and Rambouillet hospitals.

Dog*	Females	Males	≤ 50 years	> 50 years	Foch Hospital	Rambouillet Hospital
Leyko (87/92)	84/94	90/91	86/91	90/95	100/88	90/100
Jinko (89/87)	87/88	90/85	86/86	90/89	84/71	91/88
Ortie (60/90)	65/88	55/92	59/92	62/88	74/88	60/92
Oska (91/85)	90/88	93/82	95/85	89/85	88/76	100/83
Oxmo (94/91)	90/90	97/92	95/91	92/91	95/82	90/92
Ouija (90/91)	87/90	94/92	86/91	92/91	100/88	80/83
Joye (89/78)	90/78	87/78	82/81	92/74	95/88	80/67

*Overall sensitivities/specificities from Table 3 are provided for each dog in parentheses. Numbers are expressed as sensitivities/specificities.

**Table 6:** Sensitivities and specificities for the seven dogs for patients without clinical signs, past or current medical conditions and not using the most frequently reported drugs.

	Absence of clinical signs					Absence of past or current diseases		Not using drugs				
Dog*	Dyspnoea	Fatigue	Fever	Dry cough	Muscular pain	Hypertension	Diabetes	Analgesics	Anti-coagulant	Anti-hypertensive drugs	Anti-inflammatory drugs	Antibiotics
Leyko (87/92)	93/93	89/92	89/92	86/92	93/92	85/93	85/92	90/93	88/93	85/93	86/93	85/93
Jinko (89/87)	90/88	88/86	89/86	90/87	91/87	93/87	85/87	94/85	90/88	89/87	88/88	85/86
Ortie (60/90)	52/91	61/91	66/91	58/90	63/91	68/91	57/91	62/91	62/91	62/91	56/90	56/91
Oska (91/85)	89/86	94/86	91/86	94/86	98/87	95/85	88/86	94/83	89/86	96/83	89/84	89/86
Oxmo (94/91)	93/92	91/91	94/91	94/91	95/91	95/92	91/90	96/93	95/92	96/91	92/92	92/91
Ouija (90/91)	90/91	85/91	91/92	94/91	88/91	90/91	87/91	92/91	93/90	91/90	92/91	86/88
Joye (89/78)	79/78	88/79	89/79	86/78	86/78	88/81	85/77	88/80	90/78	89/79	86/79	90/77

*Overall sensitivities/specificities from Table 3 are provided for each dog in parentheses. Numbers are expressed as sensitivities/specificities.

After restricting to hospitals, sensitivity was close to the overall sensitivity, but may lack accuracy due to the small number of COVID-19 positive patients in each hospital. Sensitivity calculated after restricting to patients without diabetes were slightly lower than the overall sensitivity, although all but one remained equal or higher than 85% (57% for Ortie). Specificity remained virtually the same after the previously listed stratifications (Tables 5 and 6). The only exception was slightly lower specificity after restricting to patients recruited from Foch hospital, but this was not the case after restricting to Rambouillet hospital.

Positive and negative predictive values for each dog with infection probability ranging from 10% to 50% are shown in Table 7. The negative predictive values for all but one dog (Ortie) for a sample with ≤40% infection probability were ≥91% and almost the same as the reference diagnostic tool (97%). Three dogs maintained a good negative predictive value of ≥90% when the sample had >50% infection probability, which is close to the reference diagnostic tool (95%). Positive predictive values were good but to a lesser extent. When the infection probability was low (10%), the positive predictive values ranged from 40% to 55% for all dogs compared with 68% for the reference diagnostic tool. As expected, positive predictive value increases with increased infection probability and could be considered good (≥ 90%) for three dogs (Leyko, Oxmo, and Ouija) when the infection probability reaches 50%.

**Table 7:** Positive and negative predictive values for the seven dogs according to infection probability (ranging from 10% to 50%) based on clinical signs and/or prevalence rate in the area where the patient lives and works.

	10%		20%		30%		40%		50%	
Dog or almost perfect diagnostic tool*	PPV	NPV	PPV	NPV	PPV	NPV	PPV	NPV	PPV	NPV
Almost perfect diagnostic tool (95/95)	68%	99%	83%	99%	89%	98%	93%	97%	95%	95%
Leyko (87/92)	55%	98%	73%	97%	82%	94%	88%	91%	92%	88%
Jinko (89/87)	43%	99%	63%	97%	75%	95%	82%	92%	87%	89%
Ortie (60/90)	40%	95%	60%	90%	72%	84%	80%	77%	86%	69%
Oska (91/85)	40%	99%	60%	97%	72%	96%	80%	93%	86%	90%
Oxmo (94/91)	54%	99%	72%	98%	82%	97%	87%	96%	91%	94%
Ouija (90/91)	53%	99%	71%	97%	81%	96%	87%	93%	91%	90%
Joye (89/78)	53%	99%	50%	97%	81%	96%	87%	93%	80%	88%

*Overall sensitivities/specificities from Table 6 are provided for each dog in parentheses. Numbers are expressed as sensitivities/specificities. PPV: Positive predictive value; NPV: Negative predictive value.

## Discussion

This validation study aimed to determine the individual sensitivity and specificity values for the seven participating dogs. We found sensitivity ranged from 87% to 94% for six of the dogs and was above 90% for three dogs. Specificity results range from 78% to 92%, six dogs being above 85% and four above 90%. Our results were not affected by patient gender, age (over or under 50 years old) or by the hospital sampling site (Table 5). Furthermore, participants without associated clinical signs, concurrent conditions (diabetes, hypertension) or not taking medication do not impact the results (Table 6). These results validate the acuity of the data obtained in our previous publication [23].

The positive and negative predictive values for each dog if they were used for mass detection in areas with infection probability rates ranging from 10% to 50%, compared with a reference diagnostic test (95% sensitivity and specificity), are shown in Table 7. This reveals that while the positive predictive value of the dog is lower than the reference test, the negative predictive value is similar. The canine olfactory test therefore appears to be more accurate than RT-PCR tests, for which the false negative rate can range from 2% to 30% [37].

Our sensitivity and specificity results appear consistent with those from other sweat sample studies including Jendrny et al. [26], Eskandari et al. [28], Bjorkman et al. [38], Sarkis et al. [39] and Grandjean et al. [40]. Essler et al. [29] obtained more mixed results when using urine samples and then saliva samples, deactivated by detergent, whereas Vesga et al. [27] used respiratory secretions and saliva revealing sensitivity results ranging from 90.4% to 97.7% and specificity results ranging from 99.5% to 99.8%.

In March 2021, the World Health Organisation [41] provided a comprehensive summary of the COVID-19 olfactory detection results obtained in several countries. However, the results in this summary use the nasopharyngeal RT-PCR as a reference despite having variable results. Axell-House et al. [42] evaluated over 200 molecular diagnostic tests approved by the Food and Drug Administration (FDA) in the USA and concluded that many studies lack solidity. Zhang et al. [43] confirmed this finding and added that clinical and public health decisions cannot be solely based on RT-PCR tests. For example, Arevalo-Rodriguez et al. [44] revealed a 54% false-positive rate from RT-PCR tests in a meta-analysis of nearly 13000 patients from 34 different studies.

For a RT-PCR to be reliable, it is dependent on having enough viral genetic material in the test sample. Cycle threshold (Ct) is the number of cycles required to increase viral RNA to detectable levels. In the USA, the Centre for Disease Control and Prevention (CDC) recommends using a Ct under 30 because the false positive rate is very low below this level [45]. However, since the summer of 2020, most tests have a Ct of 40 thus increasing the false positive rate [37]. Surkova et al. [46] found that false positives can also come from various technical issues including external contamination during sampling and reagent contamination. Currently, the false positive rate could be over 4% in the United Kingdom [47], and even higher in the USA [48]. Furthermore, Kanji et al. [37] demonstrated that the false negative rate can reach 30%. This phenomenon appears to be accentuated as the COVID-19 prevalence decreases in the population [49].

In addition to related public health and ethical issues, these factors have also hindered our proof-of-concept work [23] since reliable results from the nasopharyngeal PCR analysis are needed for training dogs in olfactory viral detection. The significant number of false negatives and the increasing false positive rate as prevalence decreases complicate this training. Problems include dogs not showing a positive alert response for samples that were identified as positive and having to include new criteria which consider PCR Ct values for future samples used to train new dogs. We have therefore narrowed the positive sample inclusion criteria for the training stage (pathognomonic clinical symptomatology, and PCR Ct of ≤30) and routinely request repeat PCR tests when the dogs show a positive alert response for a negative sample.

Moving into the operational stage with six of the seven dogs tested in this study should allow more specific analysis of the possible interferences between concurrent medical conditions (including obesity, diabetes, hypertension, and respiratory diseases) and positive canine olfactive COVID-19 tests.

The strengths of this study include using different samples for training and validation, randomised sample position, one sweat sample per individual, samples only being sniffed once by each dog, double-blind study design and a large number of recruited individuals (n = 218). However, this study does present its own limitations. The number of COVID-19 positive samples (n = 62) prevented us from estimating sensitivities with accuracy, especially after stratifications on potential confounders. Increasing this number was however not possible due to the situation at the time, as it would have been too much work for the centres involved. Furthermore, COVID-19 positive and negative samples were only matched on hospital. Samples had to be sniffed within days making it impossible to wait for an appropriate matched sample, this may have created confounding bias. However, stratification (on age and sex) and restriction (hospital, clinical signs, and drugs) during statistical analysis should have accounted for this to a certain degree. Specifically, if a characteristic was a confounding factor causing high sensitivities and specificities, stratification or restriction to one characteristic would reduce sensitivity and specificity for all seven dogs, but this was not the case. Additionally, many positive samples obtained through RT-PCR had a high Ct (over 40) meaning accuracy was significantly altered so they could not be used. Another limitation is the heterogeneity of the COVID-19 negative individuals. Although these individuals presented a negative RT-PCR test at the time of inclusion, some may have had a positive RT-PCR test a few days or weeks later. Misclassification errors like this have a negative impact on specificity values. Therefore, without such misclassification errors, the specificities would have been higher than those estimated in our study. One solution to limit the impact of false positives and false negatives in COVID-19 detection dog studies would be to have a maximum acceptable Ct number to identify COVID-19 positive individuals. Furthermore, COVID-19 negative but symptomatic individuals could be included based on a negative RT-PCR test performed one or two weeks after the onsets of symptoms.

Our goal was to validate the acuity of the data obtained in our previous publication [23]. This work is an essential step before considering a study examining how the dogs respond in the presence of other viral diseases, particularly respiratory diseases. There are further studies we also plan to conduct. These include focusing on asymptomatic or subclinical cases of COVID-19 since these are the individuals most likely to spread infection, and simplifying the operational set-up by reducing contact duration with the swabs and using olfactory test lines that are faster to set-up and clean.

During our study, all seven dogs showed a confident positive alert response for several negative samples. Subsequently, a repeat nasopharyngeal RT-PCR test was performed on each patient and were positive. In three of these cases, the patients had returned to the hospital several days after the canine olfactory test, presenting COVID-19 respiratory or digestive symptoms. As Essler et al. [29] commented, dogs appear to detect the SARS-CoV-2 infection earlier than RT-PCR. Considering this finding, we are also planning a chronological approach based on the SARS-CoV-2 latency period.

## Conclusion

In our study, seven dogs successfully detected SARS-CoV-2, with sensitivity ranging from 87% to 94% for six dogs and specificity ranged from 78% to 92%. Canine detection could be a useful testing option as it is non-invasive, inexpensive compared to dedicated laboratory equipment, and provides immediate results. Remaining research challenges include optimal training methods and their standardisation for large numbers of detection dogs, and infrastructure supporting their deployments.
